# Supplementary material for: Research progress on the effects of postharvest storage methods on melon quality
Source: PeerJ. 2024 Aug 5;12:e17800. doi: 10.7717/peerj.17800 (PMC11637005; doi:10.7717/peerj.17800)
Supplement: Supplemental Information 1 — Aje company provided modifying proof of success [file peerj-12-17800-s001.pdf]

Editing Certificate

This document certifies that the manuscript

Research progress on effects of postharvest storage quality of melon

prepared by the authors

Haofei Wang, Jiayi Cui, Rui Bao, Hui Zhang, Zi Zhao, Xuanye Chen, Zhangfei Wu, Chaonan Wang

was edited for proper English language, grammar, punctuation, spelling, and overall style by one or more of the highly qualified native English speaking editors at AJE.

This certificate was issued on **June 8, 2024** and may be verified on the AJE website using the verification code **FD62-7D1C-1FD4-D86B-F82P**.

Neither the research content nor the authors' intentions were altered in any way during the editing process. Documents receiving this certification should be English-ready for publication; however, the author has the ability to accept or reject our suggestions and changes. To verify the final AJE edited version, please visit our verification page at aje.com/certificate. If you have any questions or concerns about this edited document, please contact AJE at support@aje.com.

AJE provides a range of editing, translation, and manuscript services for researchers and publishers around the world. For more information about our company, services, and partner discounts, please visit aje.com.
